# The University of London and the South Kensington Institute

**Published:** 1907-07-13

**Authors:** 


### The Hospital

A Journal of

The Medical Sciences and Hospital Administration.

New Series. No. 16, Vol. I. [No. 1088, Vol. XLII.] SATURDAY, JULY 13, 1907.

### The University of London and the South Kensington Institute.

At the meeting on Friday, June 28, the Faculty of Medicine of the University of London again expressed an opinion adverse to the proposed Institute of Medical Sciences at South Kensington.

It will be remembered that, at the recent Senatorial Election, Drs. Hill and Caley were elected representatives of the Faculty in place of Professor Rose Bradford and Dr. Kingston Fowler, the election having been fought solely on the question of the proposed Institute. It was considered desirable that in view of this election the situation should be reconsidered by the Faculty, and a special meeting was summoned for the purpose, at which there was an attendance of about 150 members. A resolution was moved by Dr. Leonard Hill to the effect that in view of the inadequate response to the appeal for the proposed Institute of Medical Sciences, of the incorporation of University College, the impending incorporation of King's College, and the published decision of four of the medical schools not to participate in the scheme of concentration at South Kensington, the Faculty was of opinion that it was no longer desirable to proceed with the scheme for the institution of a third centre for the teaching of Preliminary and Intermediate Medical Sciences at South Kensington. This was seconded by Dr. Caley, and after considerable discussion was carried by the narrow but effective margin of 77 to 72. The debate, though it never reached a high level, was instructive as crystallising, in a manner which has not been done before in public, various conflicting ideas with reference to the particular scheme which has lately been adopted by the University as its policy.

It was evident from the speeches of Dr. Norman Moore, of St. Bartholomew's Hospital, and Dr. Turney, a former Dean of St. Thomas's, that, as has been previously mentioned in The Hospital, the original scheme which attracted very many members of the Faculty of Medicine of the reconstituted University was that of a single institute, which should be neutral to all medical schools, and the erection of which would have been followed by the abandonment of the teaching of Preliminary and Intermediate Medical Studies at both University and King's Colleges.

The present opposition to the Institute at South Kensington is the direct result of the Senate having so far departed from this original idea that University College was allowed to be incorporated as a school of the University with the express stipulation in its Act of Incorporation that the teaching of Preliminary and Intermediate Medical Studies should be retained. This has altered the whole complexion of the situation, and it was very evident from the speech of Dr. Turney, who supported Professor Starling in an amendment recommending the University to proceed with the scheme, that University College and King's, from their intimate and long-standing association with hospitals, could never be regarded seriously as possible centres by medical schools which might wish to abandon their early teaching. The staff of St. Thomas's Hospital were, therefore, in favour of the South Kensington Institute being proceeded with, as they wish to relieve themselves of a certain amount of teaching which is proving a heavy financial burden to them, and they cannot do so until a neutral institute such as that proposed at South Kensington is provided.

Dr. Spriggs, representing St. George's Hospital Medical School, which has already "concentrated," made a clever speech from its point of view, and informed the Faculty that St. George's Hospital had only consented to send its students to University College or King's on the strength of an assurance from the University that the Institute of Medical Sciences at South Kensington was part of the fixed policy of the University, and he stated that, should this Institute not be proceeded with, the University would be guilty of a breach of faith towards it. We should feel inclined to doubt whether the Senate has ever given an assurance in these terms to St. George's, but even if it did, it may, on the other hand, if it persist in the three centre scheme, render itself open to a charge of breach of faith with many other of the medical schools who have refrained hitherto from expressing any definite opinion thereon, as they, relying upon an assurance made in 1901, were expecting to be consulted before any definite scheme was adopted as the final policy of the University. However, whether the University is committed in any way to St. George's Hospital or not, it seems evident that, with two definite expressions of opinion within six months, hostile to the proposed centre at South Kensington, it will be impossible to proceed with it; the Senate has probably already realised this, for it has re-committed the consideration of the scheme to an Institute Committee, who are empowered to consult the Schools. This action, though taken somewhat late in the day, is what the University should have done before University College was incorporated, and before the idea of a single centre was abandoned. Had this course been followed, the Senate would have been saved its present rude awakening.

It seems desirable that the Faculty should at an early opportunity recommend to the Senate that, if possible, no action should be taken with reference to the concentration of medical education in the University without the Faculty of Medicine being first consulted. In this way, whether the Senate accepts the recommendations of the Faculty or not, it would at any rate be able to keep itself in touch with general opinion in the medical schools of the Metropolis in a way which it has hitherto failed to do.

